# NAVIGATING THE PANDEMIC: THE ROLE OF RESILIENCE AND NEIGHBORHOOD SUPPORT IN STRESS, ANXIETY, AND ADAPTATION

**DOI:** 10.1093/geroni/igae098.1811

**Published:** 2024-12-31

**Authors:** Afnan Balubaid, Ashley Berghoff, Kristin Litzelman

**Affiliations:** University of Wisconsin Madison, Madison, Wisconsin, United States; University of Wisconsin Madison, Madison, Wisconsin, United States; University of Wisconsin Madison, Madison, Wisconsin, United States

## Abstract

The early stages of the COVID-19 pandemic were associated with increased distress and anxiety symptoms in the general population. We sought to assess how resilience and neighborhood support were associated with stress, anxiety, and positive adaptation during the first 15 months of the pandemic. We used three waves of data from the Survey of the Health of Wisconsin’s COVID-19 Community Impact Survey (Wave 1: May-June 2020; Wave 2: January-February 2021; Wave 3: June 2021; n= 3,534 observations of 1,374 unique adults). Resilience (Brief Resilience Scale), neighborhood support (Brief Sense of Community Scale), and demographics were measured at Wave 1 and outcome variables (stress, anxiety, and positive adaptation) were measured at each wave. Linear mixed models tested independent and interactive associations of resilience and neighborhood support with the outcome variables, accounting for repeated measures. Average levels of anxiety were highest at the start of the pandemic (spring 2020), while stress was highest in winter 2021 and positive adaptation was highest in summer 2021. Across waves, there was a significant interaction between resilience and neighborhood support, such that neighborhood support was more strongly associated with lower stress and anxiety in those with low resilience than high resilience (slope for stress = -0.28 vs 0.03, respectively, p[interaction]< 0.05). Resilience and neighborhood support showed small associations with positive adaptation (beta = 0.11, p<.01 and beta = 0.04, p<.01, respectively). The findings suggest both psychological and social resources buffered adverse effects of the pandemic on well-being, with implications for preparing communities for future crises.

